# Effects of stimulus duration and vowel quality in cross-linguistic categorical perception of pitch directions

**DOI:** 10.1371/journal.pone.0180656

**Published:** 2017-07-03

**Authors:** Si Chen, Yiqing Zhu, Ratree Wayland

**Affiliations:** 1 Department of Chinese and Bilingual Studies, The Hong Kong Polytechnic University, Hong Kong SAR, China; 2 Department of Linguistics, University of Florida, Gainesville, Florida, United States of America; Northwestern University, UNITED STATES

## Abstract

We investigated categorical perception of rising and falling pitch contours by tonal and non-tonal listeners. Specifically, we determined minimum durations needed to perceive both contours and compared to those of production, how stimuli duration affects their perception, whether there is an intrinsic F0 effect, and how first language background, duration, directions of pitch and vowel quality interact with each other. Continua of fundamental frequency on different vowels with 9 duration values were created for identification and discrimination tasks. Less time is generally needed to effectively perceive a pitch direction than to produce it. Overall, tonal listeners’ perception is more categorical than non-tonal listeners. Stimuli duration plays a critical role for both groups, but tonal listeners showed a stronger duration effect, and may benefit more from the extra time in longer stimuli for context-coding, consistent with the multistore model of categorical perception. Within a certain range of semitones, tonal listeners also required shorter stimulus duration to perceive pitch direction changes than non-tonal listeners. Finally, vowel quality plays a limited role and only interacts with duration in perceiving falling pitch directions. These findings further our understanding on models of categorical perception, the relationship between speech perception and production, and the interaction between the perception of tones and vowel quality.

## Introduction

Voice pitch or rate of vocal fold vibration (fundamental frequency, F0) plays distinctive linguistic roles across languages. In tonal languages such as Thai, Mandarin Chinese, Cantonese, and Vietnamese, altering the pitch level or pitch contour on a syllable or a word results in a change in its meaning or referent. For example, a Chinese syllable [ma] produced with a rising pitch contour means ‘hemp’, but the meaning changes to ‘mother’ if produced with a high-level pitch contour. In other words, Mandarin speakers could not know the meaning of the syllable [ma] when uttered in isolation without processing its associated pitch variation. On the other hand, in a non-tonal language such as English, F0 variation functions at the phrase or the sentence level to signal differences such as a statement versus a question. For instance, “A doctor” spoken with a falling F0 contour is a statement, an answer to a question such as “Who is s/he?”, whereas “A doctor?” with a rising F0 contour is a question. In both cases, the referent of the word ‘doctor’ as ‘a qualified practitioner of medicine’ remains the same, but its information status differs in terms of being an assertion or a question.

Due to the different types of meaning that tone and intonation convey, the same F0 pattern may be processed differently among tonal and non-tonal language speakers [1]. There is also consistent behavioral and neurological evidence that long-term exposure to a tone or an intonation language shape the perception of F0 differently. Native Chinese and English speakers have been found to differ on how they process level versus contour tones (e.g., [2, 3]), and how they encode pitch at the neuronal level (e.g., [4]). Overall, these studies suggest that experience with a tone language enhances pitch processing at the word level, and that tonal language speakers (e.g., Mandarin Chinese) may associate words from a non-tonal language (e.g., English) with a fixed F0 pattern.

In this study, we compared and contrasted perception of F0 contours among native Mandarin Chinese and American English speakers. Specifically, we explored the role of stimulus duration on the categorization of rising and falling F0 contours, and its interaction with vowel quality. In addition, we compare our perception results to the production results obtained by [5] to see whether the minimum duration required for pitch perception is comparable to that of pitch production by both tonal and non-tonal language speakers. Because the human articulatory apparatus obeys physical laws, the minimum duration for target movements can be measured empirically [5, 6]. In [5], native English and Mandarin speakers were asked to imitate synthesized high(H) -low(L) pitch undulation patterns (e.g., HLHLH and LHLHL). Then excursion size−pitch difference in semitone (henceforth st) between adjacent F0 minimum and maximum in the middle undulation cycle and excursion time (i.e. the time interval in between those two points) were measured (p. 1402). The scatterplots showed that excursion time varied linearly with excursion size, so they chose a simple linear regression model to model their relationship. According to [5], the mean minimum durations for rising and falling pitch contour production can be estimated using Eqs 1 and 2 below:

Rising:t=89.6+8.7*d (1)

Falling:t=100.4+5.8*d (2)

where *t* stands for time of speech production (in msec), and *d* represents the size of pitch movement in st. Specifically, they found that pitch lowering is consistently faster than pitch raising. Surprisingly, they found that native English speakers produce larger pitch excursions (i.e., pitch difference between adjacent F0 minimum and maximum in the middle undulation cycle), thus a faster pitch change, than Mandarin speakers even though the latter group has more extensive experience with producing local pitch changes in Mandarin. Despite different constraints, physical vs. auditory, placed on the production and perception systems, we wondered if the effects of L1 background and pitch direction would produce a similar pattern of results in perception.

A comparison would shed light on the relationship between production and perception. This relationship has been addressed in a number of studies. Casserly and Pisoni note a paradoxical relationship between signal variance and perceptual invariance [7]. Speakers may not necessarily perceive the differences that exist in speech production, as in the perception of Chinese third tone sandhi [8, 9]. Moreover, many scholars observe compensatory effects in the relationship between speech perception and production [10–13]. For example, syllables produced with low tones tend to be longer than those produced with high tones, but a high tone syllable is perceived as being longer than a low tone syllable [13, 14]. The current study extended this line of research by comparing the perception and production results of rising versus falling pitch contours among tonal and non-tonal language speakers.

### Tone processing and categorical perception by tonal and non-tonal language speakers

Early studies show that tonal (English) and non-tonal (Cantonese and Mandarin) language speakers assign different perceptual weights to pitch dimensions. English listeners attend more to average F0 and F0 height than F0 direction, but Mandarin listeners rely more on F0 direction than average F0 and F0 height [2, 15]. Language experience with intonational versus tonal categories [16, 17] also leads to differences in tone processing due to non-tonal language speakers’ lack of experience in establishing tonal categories. However, they may not be at a total disadvantage compared to tonal language speakers in learning a new tone language [18, 19].

Categorical perception is a phenomenon in perception where within-category stimuli are harder to discriminate than those belonging to two separate phonetic categories [20–22]. It is characterized by a sharp category boundary, a discrimination peak at the category boundary, and a strong relationship between identification and discrimination [21–25]. The strongest version of categorical perception asserts that listeners’ categorical identification completely predicts their discrimination. However, empirical results rarely support the strong relationship between identification and discrimination. With additional reaction time and goodness ratings measures, listeners can perceive differences between members of the same category. Stimuli near the category boundary usually lead to a long reaction time and lower goodness ratings [26]. The effects of fine, within- category detail in speech perception have also been documented [27, 28]

Nonetheless, a recent study reported a stronger correlation between predicted and obtained discrimination among Mandarin listeners in comparison to Dutch listeners [29]. Mandarin listeners were found to have a higher degree of categorical perception than Dutch listeners [29], based on categorical perception index computed from correlation coefficient between the classification and discrimination function [30].

Studies of categorical perception have been conducted on both segmentals [7, 21, 31–33]; and suprasegmentals [23, 34–37]. In studies on segmentals, categorical perception occurs more in perception of consonants than vowels in both behavioral and neurophysiological studies [7, 20, 21, 38, 39]. In suprasegmentals, categorical perception was found in perception of a continuum from high-rising to high-level tones in Cantonese [34] and perception of a continuum from rising to level tones in Mandarin [36,37], but not on level tones in Thai or Cantonese [34, 40]. Categorical perception of vowels and pitch directions will be further discussed in later sections.

Moreover, perception differs for listeners with different linguistic backgrounds. Burnham and Jones found that Thai listeners perceive artificial speech continua of tones more categorically than English listeners, but they are equivalent in perceiving non-speech continua, suggesting that categorical perception of tones might be related to language experience, and can be learned to some extent [41]. Hallé et al. demonstrate that Taiwan Mandarin listeners perceive tones in a quasi-categorical way, whereas French listeners’ perception is more psychophysically based [35]. In addition, Peng et al. demonstrate that, unlike tonal language listeners (Mandarin and Cantonese), the identification boundary widths are broader for non-tonal language listeners (German), whose discrimination boundaries are psychophysically- rather than linguistically determined [23].

Experience with tonal languages decreases sensitivity to the absolute pitch difference [42–44], since speakers of tonal languages make phonological category decisions on F0 continua. Moreover, neurophysiological studies also show that tone language experience can strengthen categorical neural organization to process pitch [45, 46]. One aim of this current study is to examine the effects of linguistic background on the categorical perception of rising and falling pitch continua among native Mandarin and American English listeners. Based on previous research just discussed, a stronger categorical perception is anticipated among Mandarin than among American English listeners. We also include in our investigation the roles of stimulus duration and vowel intrinsic F0, introduced in the next two sections.

### Effects of stimulus duration in speech perception

Early findings that consonant perception, particularly stop consonants, is more categorical than vowel perception suggested a role of stimulus duration in categorical perception, since consonants are generally shorter than vowels. Listeners can discriminate between two acoustically different stop consonants only to the extent that they can identify them as different stimuli [21, 32, 33, 39, 47–50]. In addition, Fujisaki and Kawashima found that short-duration (50ms) vowels are perceived near categorically [50]. However, Pisoni found that both short (50ms) and long (300ms) vowels may be discriminated in a categorical-like mode [51]. Nonetheless, categorical discrimination in vowels is different from that of consonant. Unlike vowels, consonant discrimination functions remain predictable from categorization regardless of discrimination tasks used (i.e., AXB vs. 4IAX; see [51]).

The contrasting results between vowel and consonant perception led to the proposal by Fujisaki and Kawashima of the dual-process model for the discrimination of speech stimuli [48, 50, 52]. In contrast to the modular view that perception of a given phonetic distinction is either categorical or continuous, this model assumes that two modes of perception, namely phonetic and auditory, can be active concurrently or in quick succession. The phonetic mode is categorical, and reflects phonetic classification and the associated verbal short-term memory. It is also responsible for the categorical judgment part of performance, whereas the auditory mode is continuous, and represents processes common to all auditory perception, including auditory short-term memory. Also, the auditory mode encodes into memory the acoustic properties of the stimulus, and is related to the non-categorical aspect of performance. According to this model, the difference between categoricalness of (stop) consonants and vowels is due to the differential strength of their representations in auditory memory [25]. Furthermore, auditory short-term memory may contribute to within-category vowel discrimination more than consonant discrimination [32]. Duration is purported as one possible acoustic dimension affecting representation strength. According to the cue-duration hypothesis [50], critical information in the signal of consonants, such as formant transitions, is short and may not be strongly represented and retained well in auditory memory in comparison to a longer duration, steady-state formant characteristics of isolated vowels.

Alternative to the dual-process model, in which categorical perception is hypothesized to derive from the phonetic component and continuous perception from the auditory component of the model, is the proposal that categorical perception is purely a phenomenon of auditory perception [32, 53]. According to this psychoacoustic view, some auditory dimensions of speech may not be continuous, and that the phonetic category boundaries may simply reflect innate psychoacoustic thresholds between two perceptually distinct categories on a speech continuum. For instance, the innate auditory discontinuity on a VOT continuum may influence perception of plosive consonants [33].

The possibility that perception of consonants and vowels may involve different types of memory has also been proposed [49]. Specifically, three types of memory have been discussed: a fast-decaying, sensory auditory memory; a slower-decaying, context-coding auditory memory [54]; and a long-term phoneme memory. Consonant perception involves sensory auditory and phoneme memory, which mediates contrast effects, whereas vowel perception is more affected by context-coding memory based on the range of stimuli presented in an experimental session. In addition, some mathematical models have also been proposed to explain the observed differences in perception of vowels and consonants [55, 56].

Ainsworth [57] shows that the identification rate of synthetic vowels is closely related to duration, since there are significant differences in recognition scores between “normal” duration and short vowels, as well as between long and short vowels. Meister and Werner also propose that in categorizing vowels, the openness of vowels correlates with duration among Finnish and Estonian listeners [58]. Finnish and Estonian are quantity languages, where vowel duration is exploited in phonemic duration oppositions such that a contrast of short and long vowels leads to lexical contrasts. Therefore, vowel quality is usually considered to be unrelated to vowel duration. However, a correlation between vowel quality and duration has been reported. Specifically, the high-mid vowel pairs in both of these languages show a positive correlation between vowel openness and stimulus duration. Moreover, Eerola et al. found that vowel duration may affect the categorization of Finnish vowels to some extent, as suggested by different reaction times [59]. Listeners were asked to categorize the /y/-/i/ vowel continua with different stimulus durations. Normalized reaction times demonstrate that categorization is the most difficult for vowel duration of 100ms, falling between a typical short and long Finnish vowel.

Furthermore, the perceived vowel duration is closely related to the F0 contours they bear [12, 60, 61], where falling, rising, and falling-rising F0 contours may lead to perception of longer duration than vowels bearing level F0 contours. Yu also proposes that both F0 slope and F0 height influence perception of duration, where syllables with dynamic F0 are perceived as longer than syllables with flat F0 when the acoustic duration is equal, which is congruent with production results [13]. However, syllables with higher F0 are perceived as longer than lower F0, which is the opposite of the production results and thus suggests a compensatory mechanism. Yu further confirms these findings and proposes that (i) syllables with a rising contour are perceived as longer than those with a falling contour (R > F); and (ii) syllables with rising and falling contours are perceived as longer than those with level tones (R, F > L) [62]. More importantly, he highlights an interaction between the influence of vowel height and tone on perceived duration: across tone height (high vs. low) and across dynamic tones (rising vs. falling), their effects are reduced in shorter syllables, whereas across vowel height ([a] vs. [i]) and across tonal contour (contour vs. level), their effects are lessened in longer syllables [62]. Moreover, Lehnert-Lehouillier points out that the reported impact of F0 patterns on length perception may be dependent on language background [63].

The above studies show duration as a key factor in vowel perception, which may interact with tones. In this study, we examine the role of stimulus duration in pitch perception among tonal and non-tonal listeners to better understand the roles of auditory and phonetic coding in pitch perception.

### Intrinsic F0 effect on tone perception

Intrinsic F0 effects refer to the correlation between F0 values and the vowel height, which are found to be consistent cross-linguistically [64]. High vowels ([i] and [u]) are usually associated with higher F0 values, whereas low vowels ([o], [a]) tend to be correlated with lower F0 values. Perceptually, the relationship between vowel quality and F0 values is reversed as compared to production, reflecting a compensatory mechanism. For every vowel of the same F0, low vowels /a/ and /æ/ are perceived to have a higher pitch than high vowels /i/ and /y/ in both monophthongs [11] and diphthongs [10]. Our study also aims to test whether the intrinsic F0 effect is found in categorical perception of pitch directions. The vowels /i/ and /a/ are chosen for this purpose as their height are maximally distinct.

### Current study

The current study has three main research goals: 1) to determine minimum duration needed to perceive pitch directions in comparison to production results obtained by [5] among tonal and non-tonal language listeners; 2) to examine the effects of stimulus duration on the categorical perception of pitch directions among the two groups of listeners; 3) to investigate the relationship between intrinsic F0 (henceforth IF0) effect and stimulus duration on pitch perception.

Regarding the first aim, we wonder if non-tonal language speakers would need shorter time to perceive pitch directions than tonal language speakers since they can produce them faster [5]. On the other hand, speech perception may be more efficient than speech production, thus, a shorter duration may be required for accurate perception than for production of pitch direction. In addition, a perceptual compensatory mechanism may be involved such that pitch directions requiring a longer time to produce (e.g. rising pitch) may require a shorter duration to perceive.

Our second aim attempts to answer the question of whether listeners with different linguistic backgrounds (tonal and non-tonal speakers) show different degrees of duration effects on categorical perception. In previous studies, stimulus duration was typically fixed. In our experiments, we vary stimulus duration to explore its effect on categorical perception, including the strength of categorical perception, category boundary, between- and within- category discrimination and discrimination peakedness.

**Fig 1:**
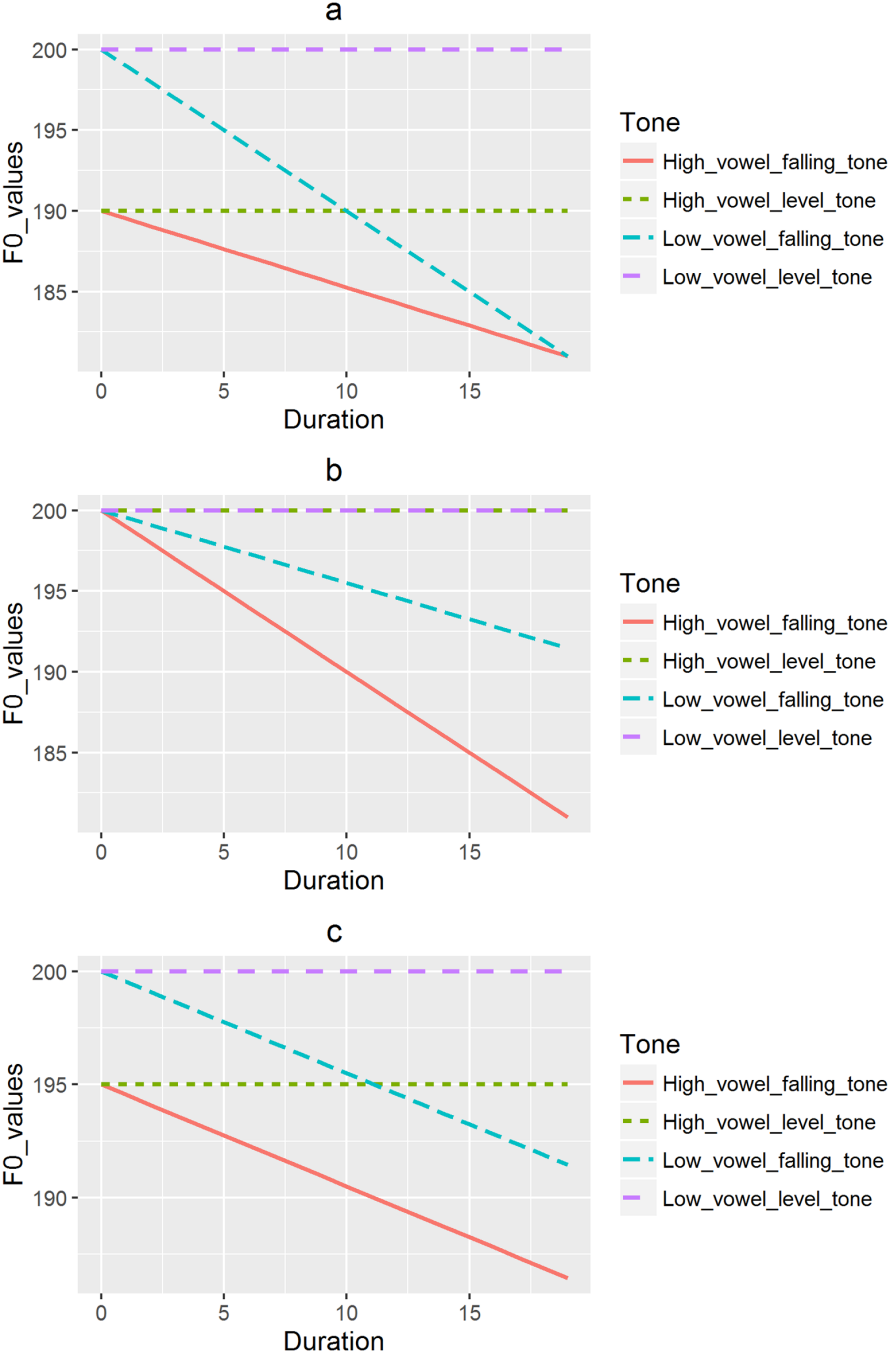
a) Falling tones on low vowels requiring a shorter duration to be perceived; b) Falling tones on low vowels requiring a longer duration to be perceived; c) Same duration required by low and high vowels.

Finally, our third aim is to investigate the relationship between duration of stimuli and vowel quality in categorical perception of pitch directions. Due to possible perceptual compensation, a falling pitch on a low vowel may require more, less or equal amount of time to be identified than on a high vowel depending on its perceived slope values. As depicted in Fig 1(a), its slope may be perceived to be steeper and more different from a level tone, and thus a shorter duration to identify if its onset value is perceived to be higher than that of a high vowel, while the perceived offset value remains the same. On the other hand, as illustrated in Fig 1(b), it may require more time to identify if its perceived slope is shallower because its offset value is perceived to be higher, while the onset value is perceived to be the same as that of the high vowel. On the contrary, as shown in Fig 1(c), its slope may be perceived to be equal to that on a high vowel, thus requiring the same amount of time to perceive if its onset and offset are equally perceived to be higher than those on a high vowel.

**Fig 2:**
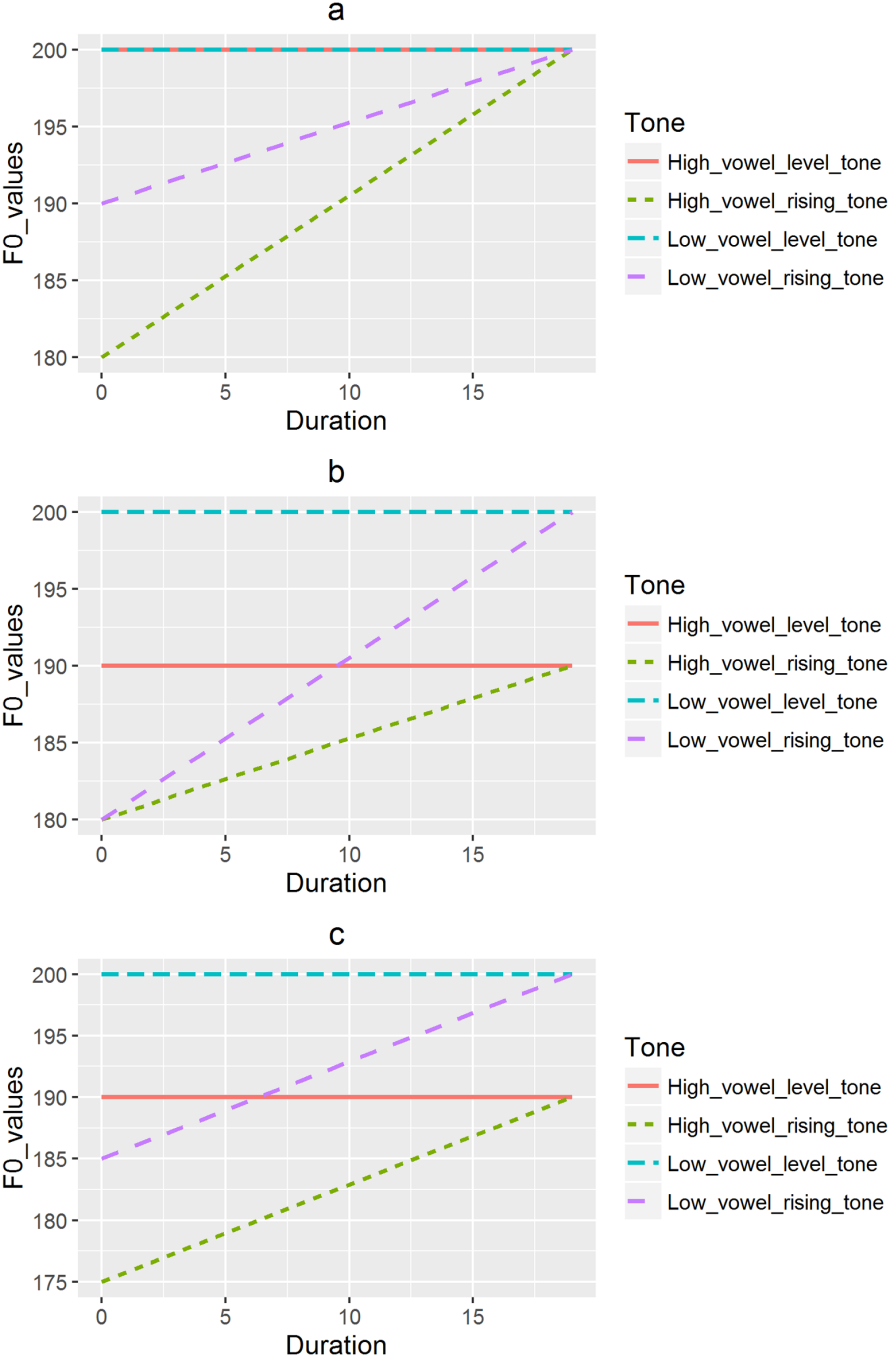
a) Rising tones on low vowels requiring a longer duration to be perceived; b) Rising tones on low vowels requiring a shorter duration to be perceived; c) Same duration required by low and high vowels.

Similar arguments may apply to rising tones. In Fig 2(a), because its onset is perceived to be higher on a low vowel relative to a high vowel, the perceived slope of a rising pitch on a low vowel is shallower and less different from a level pitch than on a high vowel, and thus a longer duration for its perception. On the contrary, in Fig 2(b), the offset of a rising tone on a low vowel is perceived to be higher, resulting in the perception of a steeper slope and a bigger difference from a level pitch, thus a shorter time for perception. Finally, Fig 2(c) suggests an equal duration required to perceive rising tones on both high and low vowels due to equal perceived slopes on both vowels.

Based on the hypothesized situations just described, the null (H_0_) and alternative hypotheses (H_a_) can be formulated as follows: 1) H_0_: the duration needed to perceive pitch directions on high and low vowels is the same; 2) H_a_: the duration needed to perceive pitch directions on high and low vowels is different.

The dual-process model, the cue-duration hypothesis, the alternative psychoacoustic view of categorical perception and various types of memory will be used to interpret and explain our results. For instance, significant differences between the two groups of listeners with different linguistics backgrounds may be explained by the dual-process model as the differences may be due to the effects of different processing modes. The cue-duration hypothesis may explain the effects of duration on category boundary location and category sharpness. In addition, contrasting effects of stimulus duration among the two groups of listeners may reflect the various types of memory involved. Finally, language-general, pyschoacoustic view may account for a lack of differential effects of stimulus duration among the two groups.

In sum, our study deepens our understanding on the factors influencing pitch perception, and achieves three research goals encompassing an investigation into duration effect, vowel quality, and linguistic experience in the perception of pitch directions.

## Methodology

### Subjects

Fifteen native Mandarin Chinese listeners (6 males; 9 females; mean age ± SD: 24.8 ± 2.83 years; age range: 21 ~ 33) were recruited from the Hong Kong Polytechnic University. On average, they received instruction in English for 11.93 years, and the standard deviation is 4.21years. The fifteen native English listeners (7males; 8 females; mean age ± SD: 20.4 ± 1.96 years; age range: 18 ~ 24) were recruited from the University of Florida to participate in this study. The participants were recruited through printed and electronic advertisements posted on campus. No participants dropped out the experiment. All participants were paid for their participation, and signed informed consent forms in compliance with a protocol approved by the Human Subjects Ethics Sub-committee at the Hong Kong Polytechnic University and the University of Florida Internal Review Board (IRB). All Chinese native speakers were from Mainland China. None of the English speakers or their parents spoke Mandarin Chinese or any other tonal languages. Only one English speaker has just started to learn a pitch-accent language, Japanese. No participants received formal instruction in Mandarin Chinese and none were exposed to other tone languages. None of the participants reported history of speaking, hearing or language difficulty. Only participants with limited music training were selected to participate, since listeners with music training may show categorical perception on a continuum [65]. None of the participants with more than five years of formal musical training or any recent formal musical training within the past five years were recruited. The two groups did not differ significantly in years of formal musical training (t(27) = 0.19, p = 0.85).

### Stimuli

#### Manipulation of stimuli

For an investigation into the factor of vowel quality, the same set of tone stimuli was created on both low and high vowels. We first recorded the Mandarin syllables [a] and [i] with a high-level tone (e.g. Tone 1: yi1, “clothing”) produced by a male native Mandarin speaker with no reported speaking or hearing problems. We recorded him using an Audio-Technica AT2020 microphone in a soundproof booth of the phonetics lab at the University of Florida.

To generate a pitch contour continuum, the pitch contour of the original target syllable was manipulated using the pitch synchronous overlap add (PSOLA) method [66] implemented in Praat. All stimuli were designed to be linear, and the slope and intercept parameters varied based on the parameters estimated from a corpus of real speech in Mandarin Chinese [67]. The estimates they proposed for the rising Tone 2 in Chinese are a slope of 93.4st/sec and an intercept of -2.2st.

The formula for calculating st from Hertz is shown in Eq (3) as follows:

$$Number\;of\;st=\frac{12}{log_{10}2}*log\left(F_{02}/F_{01}\right)$$ (3)

where *F*_*01*_ is the lower F0, and *F*_*02*_ represents the higher F0. The number of st measures the distance from *F*_*01*_ (Hz) to *F*_*02*_ (Hz) [63]. We set *F*_*02*_ to be 130Hz, the same value chosen for the offset F0 value as in [36]. Then *F*_*01*_ value was calculated based on the chosen *F*_*02*_ value and the intercept value of -2.2st as proposed in [67]. A total of 18 continua were created, one for each of the 9 durations: 200ms, 180ms, 160ms, 140ms, 120ms, 100ms, 80ms, 60ms and 40ms, for each vowel [a] and [i].

**Fig 3:**
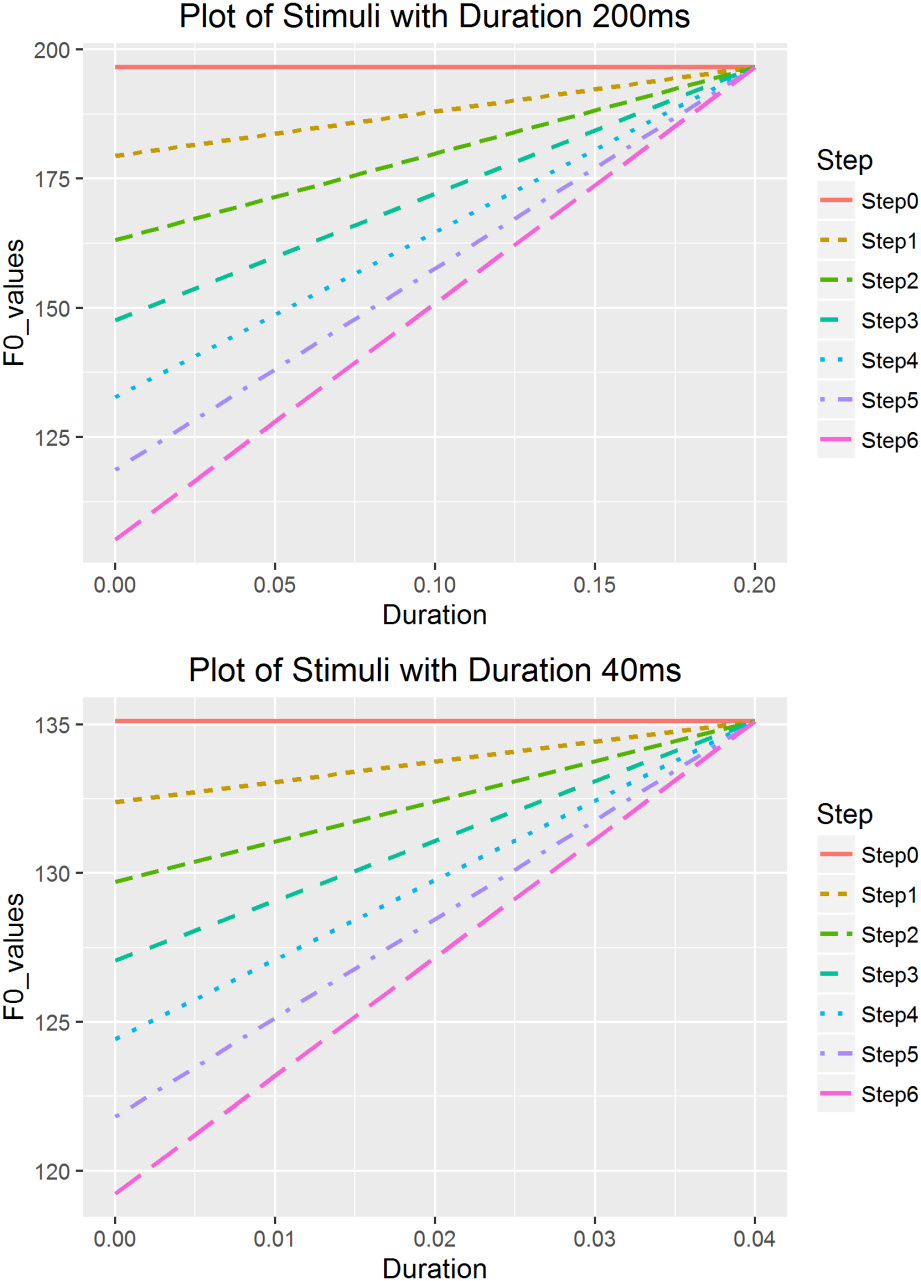
Rising stimuli in two sets of duration 200ms and 40ms.

**Fig 4:**
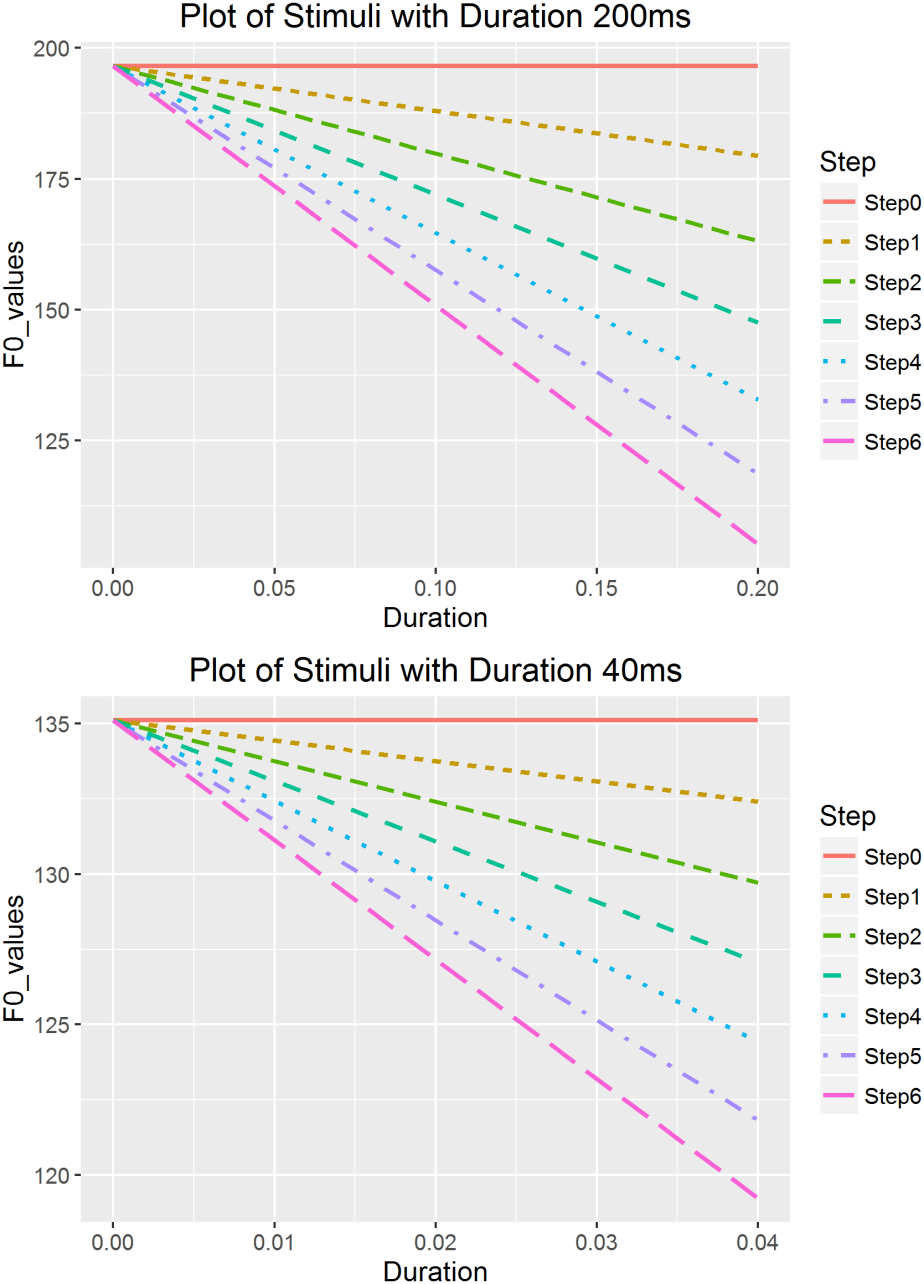
Falling stimuli in two sets of duration 200ms and 40ms.

Table 1 demonstrates all the stepwise onset values with different duration values, and Fig 3 presents a visualization of stimuli in two sets of duration 200ms and 40ms as an example. The linear falling tones were created with the reversed order of the onset values as presented in Table 2 and Fig 4. Figs 3 and 4 plotted the onset and offset F0 values for each created stimulus (step 0 to step 6) against duration (0 to 200ms and 0 to 40ms).

**Table 1:** Onset values for each step varied by duration for linear rising pitch directions.

Duration	0.2	0.18	0.16	0.14	0.12	0.1	0.08	0.06	0.04
**Step 0**	196.56	187.56	178.97	170.78	162.96	155.50	148.38	141.59	135.11
**Step 1**	179.43	172.62	166.09	159.84	153.86	148.14	142.66	137.42	132.40
**Step 2**	163.12	158.31	153.69	149.26	145.02	140.94	137.04	133.30	129.72
**Step 3**	147.58	144.61	141.76	139.03	136.41	133.91	131.52	129.24	127.06
**Step 4**	132.77	131.49	130.27	129.13	128.05	127.04	126.11	125.24	124.43
**Step 5**	118.67	118.92	119.21	119.55	119.92	120.33	120.79	121.28	121.82
**Step 6**	105.23	106.89	108.57	110.28	112.01	113.78	115.57	117.39	119.24

**Table 2:** Offset values for each step varied by duration for linear falling pitch directions.

Duration	0.2	0.18	0.16	0.14	0.12	0.1	0.08	0.06	0.04
**Step 0**	196.56	187.56	178.97	170.78	162.96	155.50	148.38	141.59	135.11
**Step 1**	179.43	172.62	166.09	159.84	153.86	148.14	142.66	137.42	132.40
**Step 2**	163.12	158.31	153.69	149.26	145.02	140.94	137.04	133.30	129.72
**Step 3**	147.58	144.61	141.76	139.03	136.41	133.91	131.52	129.24	127.06
**Step 4**	132.77	131.49	130.27	129.13	128.05	127.04	126.11	125.24	124.43
**Step 5**	118.67	118.92	119.21	119.55	119.92	120.33	120.79	121.28	121.82
**Step 6**	105.23	106.89	108.57	110.28	112.01	113.78	115.57	117.39	119.24

We use the rising continuum as an example to illustrate the specific procedure of calculating the steps. First, we calculated the offset value for each duration using the formula shown in Eq (4).

X(t)=93.4*t−2.2 (4)

where *t* stands for duration (s), and *X(t)* for the st values at the offset of the tone. For example, for the *t* = 200ms (0.2s) duration continuum, the onset can be calculated using Eq 4 by setting *t* = 0s, which is -2.2st (or 123.02Hz, using 130 Hz to transform from st to Hz). The offset can be calculated using Eq 4 by setting *t* = 0.2s, which is 16.48st (196.56Hz), and thus an onset-to-offset distance of 18.68st. Based on the calculated intercept (onset) value of -2.2st (the cutoff point), we created stimuli with various onsets. We determined the extreme points of onset values so that the distance between the lowest onset value (< -2.2st) and -2.2st was one third of the distance between the highest onset value (> -2.2st) and -2.2st. We could have set -2.2st as the median point between the lowest and highest onset values; however, we were more interested in the perception of shallower slopes close to level pitch contours, which have higher onset values. The point with a distance of one third below the cutoff point was the lowest onset, which in this case was -8.43st (105.23Hz), calculated by deducting 1/3*18.68st from the onset -2.2st. The highest onset was defined as the same value of the obtained offset value, 196.56Hz in this case, and was used to generate a level tone with equal onset and offset values.

After obtaining the highest and lowest onsets, in this case 105.23 Hz and 196.56 Hz respectively, we created steps between these values based on the ERB scale instead of Hertz, since it reflects natural perception [36]. For each duration value, we created 7 stimuli with equal perceptual distance in total. Then the calculated onset values of each step were transformed back into Hertz.

The resynthesizing procedure is similar to that described in [23]: (i) The duration of the stimuli was adjusted to the duration presented in Tables 1 and 2; (ii) The stimuli were peak normalized to the same intensity level; (iii) The number of pitch point was reduced to 2, one at the starting point, one at the end point; (iv) The pitch points were adjusted to the values presented in Tables 1 and 2 depending on duration values.

#### Stimuli used in the identification task

Stimuli were presented in two blocks in the identification task. All stimuli with a rising pitch continuum for each syllable ([a] and [i]) were presented in one block and all stimuli with a falling pitch continuum were presented in the other block. A total of 630 stimuli were presented in each block (5 repetitions*7 steps*9 duration*2 syllable). Within each block, the order of presenting all stimuli of various vowels and duration was randomized. The order of presenting all the blocks was also randomized for each subject.

#### Stimuli used in the same-different task

All stimuli in each continuum were paired for a same-different discrimination task. Since a 1-step difference is generally shown to be too difficult for listeners to perceive [23, 34], a 2-step difference was used instead. Similar to the identification task, stimulus presentation was blocked by rising and falling pitch directions. Within each block the same pair (0–0, 1–1, 2–2, etc.) and different pairs were presented in either the forward order (0–2, 1–3, 2–4, 3–5, 4–6) or the backward order (2–0, 3–1, 4–2, 5–3, 6–4). All the stimuli were repeated twice and presented in a random order, totaling 34 trials (2 repetitions * (7 same pairs + 10 different pairs)) per block. Altogether, participants heard 1224 trials (9 durations*(7 same pairs + 10 different pairs)*2 syllables*2 continua). The order of presenting all the blocks was also randomized for each subject.

### Procedure

All native Chinese listeners participated the experiments wearing a GMH C 8.100 D head set at the speech lab of the Hong Kong Polytechnic University. All native English listeners participated in the experiments using a BOSE AE2 headphone at the linguistics lab of the University of Florida. This study was designed and conducted from 2015 to 2017.

#### Training and practice tasks

Prior to the identification (ID) task, participants were familiarized with level, rising, and falling pitch directions in a training session, where sounds of each type with corresponding pictures were provided on the computer screen. Participants were trained to judge what they heard by pressing buttons, where the number key 1 stood for a level tone and the number key 2 stood for a rising/falling tone. After the training, they were tested on their ability to identify these tones using stimuli with level tones and the steepest rising or falling tones of the longest duration value (0.2s). We made sure that all recruited participants passed a minimum threshold of correct responses significantly above the chance level, determined by binomial tests with a significant level of 0.05, suggesting that they could identify pitch directions with either a level or the steepest rising or falling pitch contours of the longest duration value. Only one American participant did not meet the criterion and was excluded from the experiment.

Prior to the same-different (SD) task, the participants were also familiarized with the experimental procedure. They were asked to listen to trials containing a pair of stimuli and to decide whether the presented stimuli have the same or different pitch contour by pressing the number key 1 for “same” and the number key 2 for ‘different’ on the keyboard. After the training, a set of stimuli containing pairs of level tones and the steepest rising or falling tones of the longest duration value (0.2s) was presented to test their ability to discriminate pitch directions. Again, they had to pass a minimum threshold suggesting that they could discriminate pitch directions with either level or the steepest rising or falling pitch contours of the longest duration value to progress to the experimental trials.

These training and practice tasks aimed to familiarize participants with the experimental procedure and labels of pitch directions for stabilized performance in the real experiments. The training also helped native English speakers familiarize with Chinese syllables in order to minimize the task difficulty differences between two language groups suggested [36]. The results from the practice session were not included in statistical analysis.

#### Identification task

In the identification task, participants listened to one stimulus in each trial from one block of the rising or falling continua. They were instructed to press the same keys as in the practice session upon hearing each stimulus. All the stimuli were presented in a random order using the E-PRIME software. The participants made judgments at a self-paced rate. Once they responded to a trial, the next trial was played automatically. The collected raw identification data were transformed into a response of either 0 or 1 for further statistical analysis.

#### Same-different discrimination task

For the same-different discrimination task, participants listened to a pair of stimuli presented in each trial and decided whether the two stimuli were of the same pitch contour or not by pressing the same buttons as in the practice session. In total, there were two blocks of stimuli: one for the rising continuum and the other for the falling continuum. The order of the two blocks was also randomized for each subject. The collected raw discrimination data were transformed into a response of either 0 or 1 to fit generalized linear mixed models, and d-prime scores were also computed for further statistical analysis. The d-prime scores were calculated using Eq (5):

$$d^{\prime }=z\left(H\right)-z\left(F\right)$$ (5)

where *d’* is the d-prime score, H is the hit rate, F is the false alarm rate and *z* is z-transform [68].

One important variable that we took into consideration is the effect of interstimulus intervals (ISIs). Different ISI conditions might lead to various types of perceptual processing such as auditory, phonetic, and phonemic processing in speech perception [69]. Phonemic processing is perception based on language-specific phonological categories, and phonetic processing reflects phonetic distinctions in any language, whereas auditory processing is for perception that has no phonetic boundaries. Werker and Logan show that phonemic perception occurs at 1500ms ISI, phonetic processing at both 250ms and 500ms, and auditory processing at 250ms [69]. In this study, we used an ISI of 500ms since most studies agree that 500ms is the time needed to maximize differences in between- vs. within- category discrimination [23, 32, 36].

## Data analysis

There are three essential characteristics of categorical perception [70], which remain applicable in categorical perception research [36, 71]: sharp category boundary, corresponding discrimination peak, and prediction of discrimination from identification. In addition to analyzing these characteristics, we also examined whether linguistic backgrounds (group effect of native Mandarin and English listeners), pitch directions (rising vs. falling), vowel quality (low vs. high) and duration may contribute to pitch categorization.

### Identification task

First, we fit a generalized linear mixed model to the data using the “lme4” R package [72], including a random effect of subjects to examine if linguistic background, pitch direction, vowel quality, and duration affect identification rate of the rising and falling pitch directions.

There were eight subgroups of data based on language groups, pitch direction and vowel quality (FCA, FCI, RCA, RCI, FEA, FEI, REA, REI), where F and R stand for falling and rising pitch directions, C and E stand for Chinese and English, and A and I stand for [a] and [i] syllables. Thus, FCA stands for a subset of data consisting of falling pitch directions (F) on the syllable [a] (A) perceived by native Mandarin Chinese listeners (C).

Second, within each subgroup, we fit a generalized linear mixed model again with identification scores (0 or 1) as the response variable and step number (x = 0–6) as a factor. Recall that each stimulus continuum was created with 7 steps of equal perceptual distance for each duration value. The step number was treated as a continuous variable as suggested by [36] even though we sampled only discrete values. The model is similar to a logistic regression model when we only consider the fixed effects in Eq (6)

$$log_{e}\left(\frac{p_{1}}{1-P_{1}}\right)=b_{0}+\;b_{1}x$$ (6)

A generalized linear mixed model enables us to obtain an estimate of *b*_*1*_ and *b*_*0*_ while modeling random effects from subjects. The coefficient *b*_*1*_ stands for the sharpness of category boundary as suggested by [36, 73]. In order to perform a post-hoc analysis on the effects of sharpness of boundary by duration, we performed pairwise comparisons between different duration values, within each of the eight subgroups separately. Specifically, we fit a model treating the coefficient *b*_*1*_ from each pair of duration values as the same, and conducted a likelihood ratio test to compare with another model treating them as different. Significant differences between the two models indicate significant differences of the coefficient *b*_*1*_ between two duration values. In addition, we tested whether language group effects, pitch direction and vowel quality may affect the values of *b*_*1*_, sharpness of category boundary, and conducted post-hoc analyses. The relationship between the sharpness of category boundary and duration was also modeled for native Chinese and English listeners.

Third, after obtaining the estimates for *b*_*0*_ and *b*_*1*_, we used *P*_*1*_ = 0.5 to estimate the step number at category boundary (*x*_*cb*_) within the Chinese and English groups [36]. When *P*_*1*_ = 0.5, the equation becomes Eq (7).

$$x_{cb}=-b_{0}/b_{1}$$ (7)

Once individual category boundary was obtained for subjects in each subgroup (e.g. FCA), we fit a linear mixed effects model, including pitch directions, group effects, duration, and vowel quality as fixed effects, and subjects as random effects, and performed post-hoc analyses. We proceeded to study the relationship between duration and category boundary by fitting linear regression models separately for native Chinese and English listeners.

Finally, we obtained formulas for perception to compare with those proposed for speech production [5]: *t = b*_*0*_ *+ b*_*1*_*d*, where *t* is the duration needed for perceiving *d* st differences from level tones. For each duration value, we recorded the estimated step where the identification rate was 0.5, and set it as a cut-off point where step numbers larger than this cut-off point indicated that it was more likely not to be identified as a level tone. We transformed the step number back to st values with respect to the baseline (level tone with step 0) for each duration value. Then we fit linear mixed effects models to obtain a relationship between cut-off st values and duration, assuming that the cut-off st values were the smallest values that a rising or falling pitch direction could be perceived as different from a level tone. We obtained each formula for each subgroup (e.g. FCA), and then provided separate formulas for native Chinese and English listeners to perceive falling and rising pitch directions, as well as more general formula for perceiving each pitch direction by both groups of listeners. The intercept and slope values were also tested statistically by likelihood ratio tests.

### Same-different discrimination tasks

We obtained the correct (hits) and incorrect responses (false alarms) in the same-different task, and fit a generalized linear mixed model with three factors: duration, language group, and vowel quality, as well as all of the two-way and three-way interactions.

In the same-different task, for each duration value, we had a total of 34 trials of stimuli (2 repetitions * (7 same pairs + 10 different pairs)). We divided these trials into five 2-step comparison units (0–2, 1–3, 2–4, 3–5, 4–6), where each unit consisted of four types of comparisons (AA, AB, BA, BB). The same trials were included (e.g., the 4–4 pair was included for both 2–4 and 4–6 units). Each unit consisted of 8 trials (4 types of comparisons * 2 repetitions).

We calculated the d-prime score of between- and within- category discrimination for each subgroup according to its category boundary [71]. *P*_*bc*_ (between-category discrimination) is defined as *P* of the comparison unit corresponding to the category boundary, and *P*_*wc*_ (within-category discrimination) is defined as the average of two comparison units at the extremes of the continuum (*P*_*02*_ and *P*_*46*_) as suggested by [32]. The same calculation was done for all 9 duration values. We calculated d-prime scores for *P*_*bc*_ and *P*_*wc*_. If the category boundary was less than 1 or greater than 5 for some duration, then the d-prime score was not calculated, since the steps were constrained between 0 to 6 steps (For native Chinese listeners, the category boundary falls out of the range mainly for duration values < 80ms, but for native English listeners, < 160ms). Linear mixed-effects models were fitted to examine the contribution of language group effects, pitch direction, vowel quality, and duration to the d-prime scores, followed by post-hoc analyses. Pairwise comparison of duration was also performed, and linear regression models were fitted to examine the relationship between discrimination scores and duration.

The peakedness of discrimination function was estimated by the difference between *P*_*bc*_ and *P*_*wc*_. Linear models were also fitted to examine significant contributing factors. Pairwise comparison of duration with respect to peakedness was also conducted, and regression models were fitted to examine the relationship between peakedness and duration.

### Prediction of discrimination from identification

Pollack and Pisoni proposed the predicted discrimination score P* by calculating Eq (8):

$$P^{*}=\left[1\;+{\left(P_{A}-\;P_{B}\right)}^{2}\right]/2$$ (8)

where *P*_*A*_ and *P*_*B*_ are the identification scores in a comparison unit [74]. This equation is based on the assumption that the identification of the two stimuli A and B can solely determine the same-different discrimination [36]. Then the correlation between the predicted and the observed discrimination scores for each subgroup of each duration was calculated based on different trials of stimuli A and B by optimizing linear regression models after Fisher’s *z* transformation. Then, we tested whether the factors duration, pitch direction, language group effects, and vowel quality contributed to the correlation. The mean difference between the predicted and observed discrimination scores P-P* were also calculated. We chose the optimized linear model based on the stepwise optimization algorithm using the function “step” in [75] to model the relationship between the distance and the factors duration, pitch direction, language group effects, and vowel quality.

## Results

### Identification task

A generalized linear mixed model of identification rate suggested significant main effects of vowel quality (χ^2^(1) = 7.32, p = 0.007), language group (χ^2^(1) = 7.85, p = 0.005), pitch direction (χ^2^(1) = 22.20, p < 0.001), and duration (χ^2^(1) = 2070.80, p < 0.001). The results suggested that all the variables we tested made significant contributions to pitch direction identification rate. Specifically, a pitch direction on high vowels is more likely to be identified as different from a level one than for low vowels. Native Mandarin listeners are more likely to identify a pitch direction as rising or falling than native English listeners. A falling pitch direction is more likely to be identified as being different from a level one than a rising pitch direction. The longer the stimulus duration is, the more likely that it is identified as different (as rising or falling) from a level tone

#### Sharpness of category boundary

**Fig 5:**
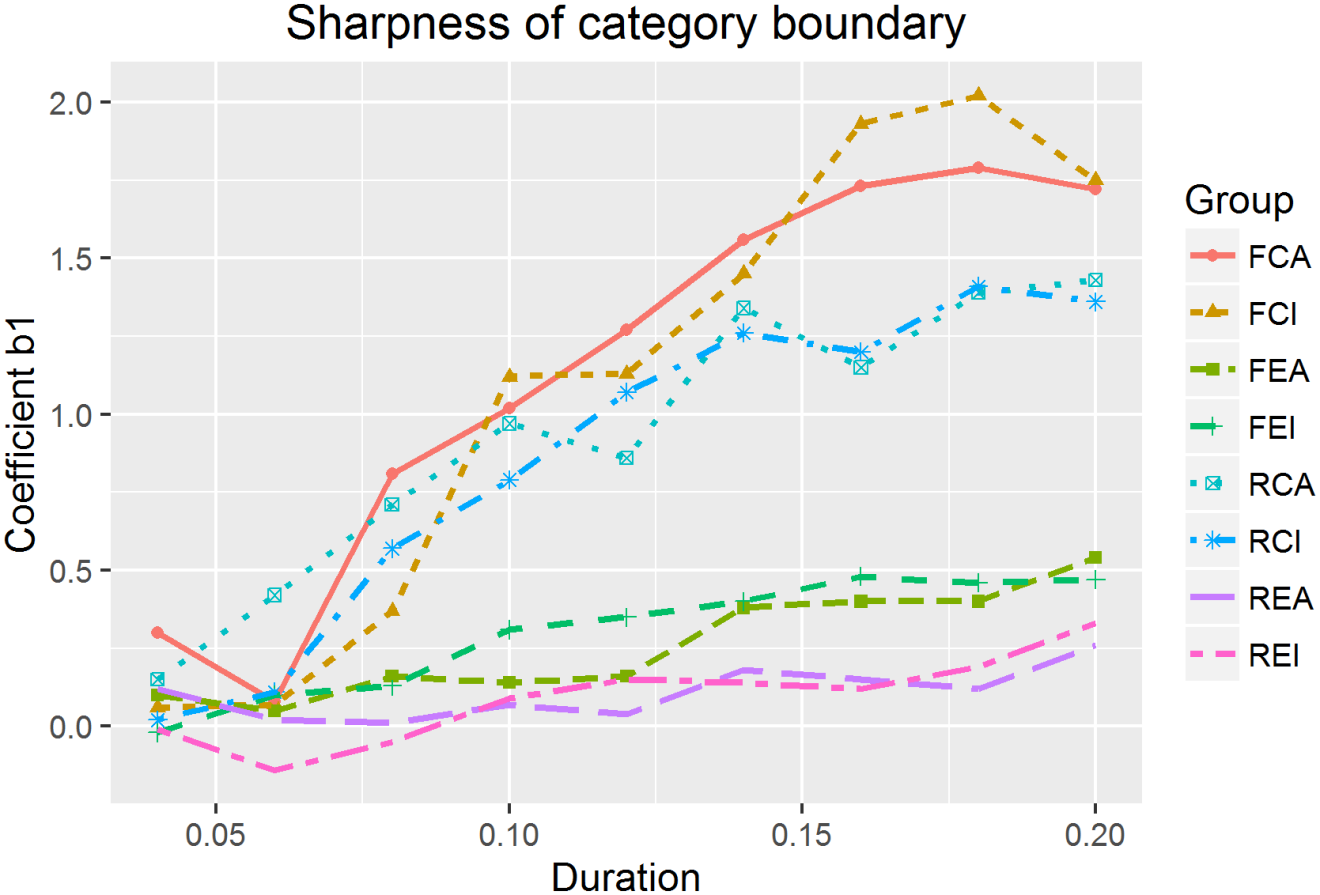
Sharpness of category boundary for each subgroup.

To examine the sharpness of category boundary with respect to duration, we first plot the estimates of the coefficient *b*_*1*_ against duration for each subgroup as shown in Fig 5. In all possible pair-wise comparisons of duration values within each subgroup (e.g. 40ms vs. 60ms; 40ms vs. 80ms), most pairs reached significance. In total, 58% of the pair with a difference of 20ms and 86% of the pair with a difference of 40ms reached statistical significance. It can be inferred that in general a duration difference of 20ms was likely to lead to a significant difference in the sharpness of category boundary, and the longer the duration, the sharper the category boundary.

In addition, likelihood ratio tests showed that Chinese and English listeners differed significantly in the sharpness of category boundary (χ^2^(1) = 1747.5, p < 0.001). Pitch direction (χ^2^(1) = 38.24, p < 0.001) also had a significant effect on the category boundary sharpness, but vowel quality did not (χ^2^(1) = 0.71, p = 0.4). Post-hoc analyses were performed to further examine language group and pitch direction effects. Native Chinese and English listeners differed in all of the four pairs (FCA vs. FEA, FCI vs. FEI, RCA vs. REA and RCI vs. REI) for most duration values. However, the effects of pitch direction were not as salient as the group effect, with significant differences between falling and rising pitch directions only at some duration values (mostly with a duration value of 40ms, 60ms and 200ms). For most duration values, the falling pitch direction elicited a sharper category boundary than the rising pitch direction. S1 Table only lists all insignificant results of all pairwise comparisons of duration values since most comparisons turned out to be significant.

Since the two groups of listeners showed significant differences in category sharpness, we fit two regression models for each group separately to examine the relationship between duration and the sharpness of category boundary. For native Chinese listeners, a regression model with an extra quadratic term better captured the relationship than the model with only the slope and intercept terms, as a likelihood ratio test suggested a significant difference (χ^2^(1) = 9.97, p = 0.002). The regression model predicted the sharpness well (F(2, 33) = 111.7, p < 0.001; adjusted R^2^ = 0.86). Specifically, the formula for the relationship between the sharpness of category boundary (b_1_) and duration (d) is in Eq (9).

$$b_{1}=\;-50.65*d^{2}+\;22.47*d-0.82$$ (9)

For native English listeners, a model with linear terms were sufficient with no significant improvement after adding a polynomial term (χ^2^(1) = 0.12, p = 0.73). The regression model reached significance (F(1, 34) = 38.26, p < 0.001; adjusted R^2^ = 0.52), and the relationship between the sharpness and duration was captured in Eq (10).

$$b_{1}=\;2.36*d-0.09$$ (10)

For both groups, sharpness of category boundary increased as duration increased, but native Chinese listeners showed a faster increment, and had a sharper boundary than English listeners for most duration values.

#### Category boundary location

**Fig 6:**
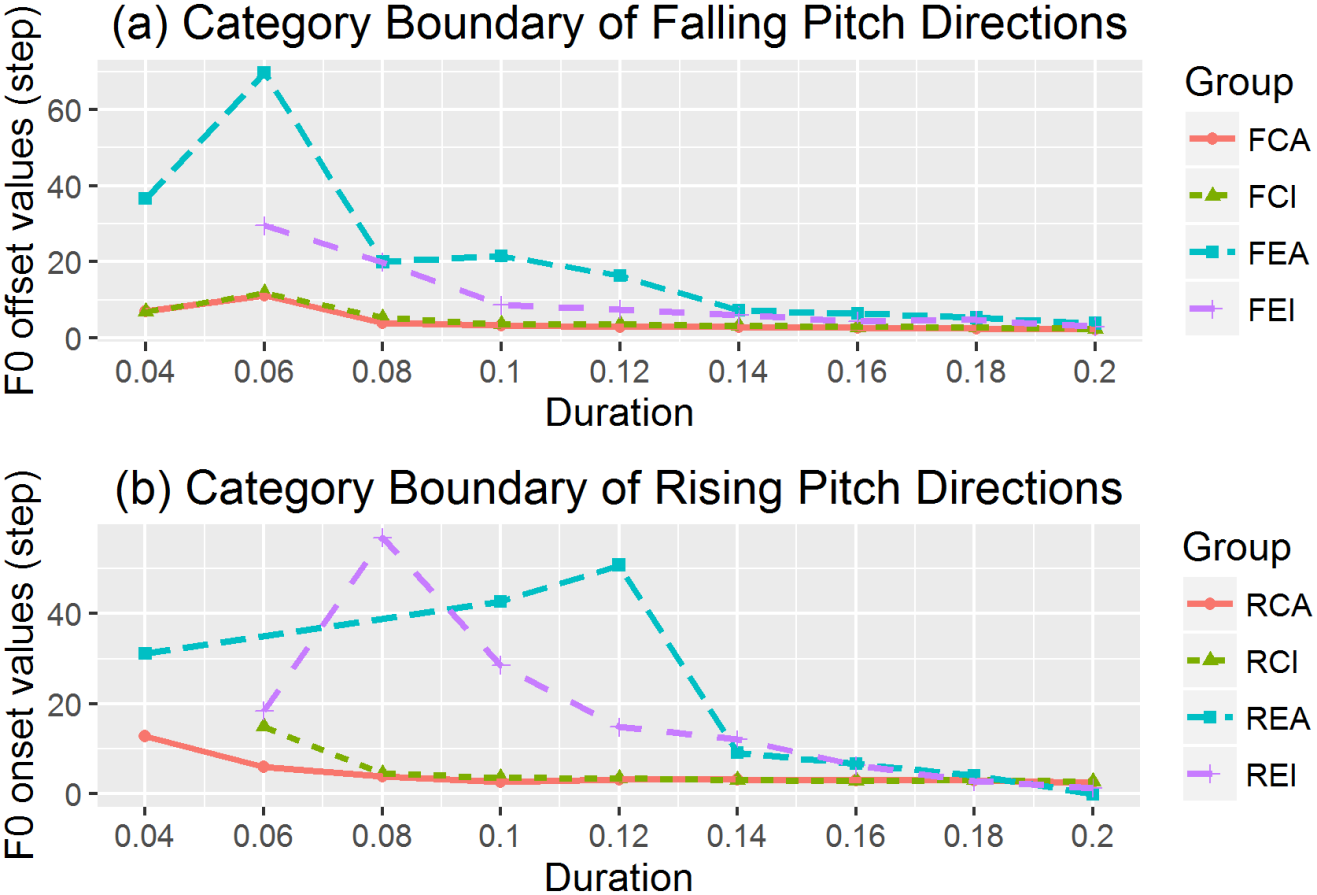
a) Category boundary of falling pitch directions; b) Category boundary of rising pitch directions.

After estimating the coefficient *b*_*0*_ and *b*_*1*_ from the generalized linear models, we calculated the category boundary for each subgroup and duration value. Fig 6 plots the category boundary against duration of stimuli for each subgroup. There were no significant fixed effects of pitch direction and vowel quality on category boundary. The two language groups differed significantly at duration values of 80ms (χ^2^(1) = 4.89, p = 0.03), 120ms (χ^2^(1) = 4.42, p = 0.04) and 140ms (χ^2^(1) = 4.28, p = 0.038). For these three durations, category boundaries occurred at earlier steps−higher offset values for falling contours and higher onset values for rising contours−for Mandarin listeners.

Since the language group effect was significantly different in some duration values mentioned above, we fit two regression models for each group to examine the relationship between category boundary and duration. For native Chinese listeners, a regression model with an extra quadratic term better captured the relationship than the model with only the slope and intercept terms, as a likelihood ratio test suggested significant difference (χ^2^(1) = 11.29, p < 0.001). Specifically, the formula for the relationship between the category boundary (*cb*) and duration (*d*) is listed in Eq 11, and the regression model was significant (F(2, 32) = 27.6, p < 0.001; adjusted R^2^ = 0.61).

$$cb=\;517.82*d^{2}-169.94*d+\;16.27$$ (11)

However, for native English listeners, a model with an extra quadratic term did not improve significantly (χ^2^(1) = 0.05, p = 0.82). Specifically, the formula for the relationship between the category boundary (*cb*) and duration (*d*) is listed in Eq 12, and the regression model was significant (F(1, 30) = 33.47, p < 0.001; adjusted R^2^ = 0.51).

cb= −258.66*d+ 50.53 (12)

For both language groups, category boundary starts earlier (i.e., at higher offset values for falling tones and higher onset values for rising tones) as stimulus duration increases, but at a slower rate for native Chinese listeners.

**Fig 7:**
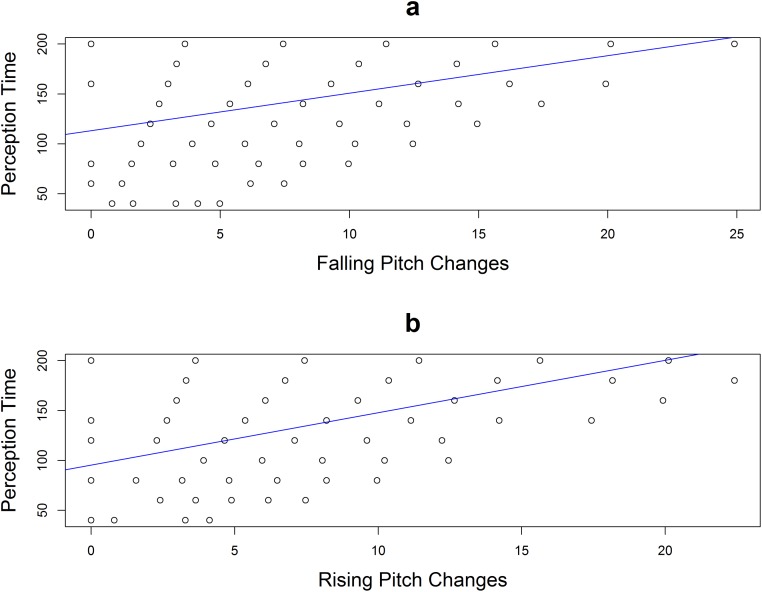
a) A plot of stimulus duration required for perception against falling pitch changes; b) A plot of stimulus duration required for perception against rising pitch changes.

Recall that Xu and Sun proposed a formula for producing a rising pitch direction: *t* = 89.6 + 8.7 * *d* and a formula for producing a falling pitch direction: *t* = 100.4 + 5.8 * *d* [5]. They obtained the formula by fitting a simple linear regression between production time and pitch changes (excursion size) since they observe a linear relationship between the two. Interestingly, similar to their results, we also observed that stimulus duration required for perception varies linearly with pitch changes (rising or falling) as shown in Fig 7. After fitting simple linear regression models, we also obtained similar R^2^ as that in production results [5] (rising pitch perception: R^2^ = 0.26; rising pitch production R^2^ = 0.2; falling pitch perception: R^2^ = 0.11; falling pitch production R^2^ = 0.10). Based on their performance on the identification task, we obtained the following formulas, where *t* is the duration needed (ms) for perceiving *d* st differences from level tones, to compare with those derived for speech production.

Rising, Chinese & American Speakers:t=95.39+5.25*d (13)

Falling, Chinese & American Speakers:t=113.27+3.75*d (14)

Compared with formulas proposed in [5], the time required to effectively perceive pitch directions are shorter than to produce them in most cases (> 1st rise or > 6st fall). In addition, across both groups of listeners, we see that a shorter duration is required to perceive a rising pitch than a falling pitch direction when the rise and fall are not greater than 11st. However, since Eq 13 has a sharper slope, 1st increment results in longer time required to perceive a rising pitch direction than a falling pitch direction.

Note that a linear relationship between duration and pitch change in both production and perception does not necessarily mean that the physical laws of human articulatory apparatus that constrain pitch production also influence pitch perception. Speech perception is a complex process, constrained by general auditory and perceptual mechanisms as proposed by the general auditory and learning approaches to speech perception [76]. Nonetheless, the fact that the relationship between production and perception time and pitch change can both be linearly modelled is of great interest. More research is needed to shed further light on the issue.

To explore the effects of language background, we derived the following four formulas for native Chinese and English listeners, where *t* is the duration needed to perceive *d* st differences from level tones.

Rising, Chinese Listeners:t=66.76+8.39*d (15)

Falling, Chinese Listeners:t=99.45+5.29*d (16)

Rising, American Listeners:t=137.33+1.89*d (17)

Falling, American Listeners:t=144.66+1.5*d (18)

From these equations, we see that non-tonal English listeners require longer time to perceive both a rising and a falling pitch direction when the rise is less than 11st and the fall is less than 12st. In addition, to explore the effects of language backgrounds and vowel quality on minimum duration required for pitch direction perception, we obtained formulas for each subgroup as follows, where *t* is the duration needed to perceive *d* st differences from level tones.

Rising, Chinese Listeners, Low Vowel (RCA):t=57.50+9.31*d (19)

Falling, Chinese Listeners, Low Vowel (FCA):t=70.69+8.84*d (20)

Rising, American Listeners, Low Vowel (REA):t=129.43+2.40*d (21)

Falling, American Listeners, Low Vowel (FEA):t=136.11+2.24*d (22)

Rising, Chinese Listeners, High Vowel (RCI):t=79.31+7.12*d (23)

Falling, Chinese Listeners, High Vowel (FCI):t=118.32+3.21*d (24)

Rising, American Listeners, High Vowel (REI):t=154.69+0.80*d (25)

Falling, American Listeners, High Vowel (FEI):t=135.36+1.70*d (26)

When we submitted the intercept and slope values to the likelihood ratio tests, we found that Chinese and American listeners have significantly different intercept and slope values (χ^2^(2) = 43.32, p < 0.001) with higher intercept values and shallower slopes for English listeners than for Mandarin listeners. The intercept values for the falling pitch are also significantly higher than those of the rising pitch, but the opposite is true for the slope values (χ^2^(2) = 6.19, p = 0.045). Both intercept and slope values are also significantly different for low and high vowels (χ^2^(2) = 10.54, p = 0.005), with the high vowel having greater intercept, but shallower slope values than the low vowel in general. These results suggest that for 1st increment, (i) across both vowels and both pitch directions, Mandarin listeners requires a longer duration to perceive a pitch direction change than American listeners; (ii) averaged across both groups of listeners and both vowels, a falling pitch requires a relatively shorter duration to perceive than a rising pitch; and, (iii), across both groups of listeners and both pitch directions, a longer duration is needed to perceive a pitch direction change on a low vowel relative to a high vowel.

Results of the post-hoc analyses presented in Table 3 show significantly higher intercept values, but shallower slope values, among native English listeners than Mandarin listeners for the falling pitch produced on the low vowel [a] and for the rising pitch produced on both the low [a] and the high [i] vowels. The difference did not reach significance for the falling pitch produced on the high vowel [i]. In addition, for Mandarin listeners, the rising pitch produced on the high vowel [i] has a significantly lower intercept value, but steeper slope value than those of the falling pitch. Finally, across both groups of listeners, intercept values for the falling pitch produced on the high vowel is significantly greater than those produced on the low vowel, but the opposite is true for the slope value.

**Table 3:** The post-hoc analyses of intercept and slope values for each pair of formulas.

Pairs of subgroups	Likelihood ratio tests
**C vs. E**	
**FCA vs. FEA**	χ^2^(2) = 7.78, p = 0.02*
**FCI vs. FEI**	χ^2^(2) = 1.36, p = 0.51
**RCA vs. REA**	χ^2^(2) = 24.41, p < 0.001*
**RCI vs. REI**	χ^2^(2) = 20.45, p < 0.001 *
**F vs. R**	
**FCA vs. RCA**	χ^2^(2) = 5.07, p = 0.08
**FCI vs. RCI**	χ^2^(2) = 7.55, p = 0.02*
**FEA vs. REA**	χ^2^(2) = 0.15, p = 0.93
**FEI vs. REI**	χ^2^(2) = 1.96, p = 0.38
**A vs. I**	
**FCA vs. FCI**	χ^2^(2) = 6.52, p = 0.038*
**RCA vs. RCI**	χ^2^(2) = 2.35, p = 0.31
**FEA vs. FEI**	χ^2^(2) = 0.45, p = 0.8
**REA vs. REI**	χ^2^(2) = 2.57, p = 0.28

F: Falling; R: Rising; C: Chinese; E: English; A: Vowel [a]; I: Vowel [i]

These results indicate that, for 1 st increment, (i) American English listeners need shorter duration than Mandarin listeners to perceive falling tones on the lower vowel [a], and rising tones on both the low vowel [a] and the high vowel [i]; (ii) Mandarin listeners require a shorter duration to perceive falling tones than rising tones on the high vowel [i]; and (iii) also among Mandarin listeners, a longer duration is needed to perceive falling tones on the vowel [a] than on the high vowel [i].

To further probe into these significant results, we calculated minimum duration needed to perceive a pitch direction change between 1 to 20 semitones rise or fall, and found that (i) English listeners need longer duration than Mandarin listeners to identify falling tones on the low vowel when the fall is ≤ 9st. The opposite is true when the fall is >9st; (iia) a longer duration is needed to perceive rising tones on the low vowel among the English listeners when the rise is ≤ 10st; and (iib) on the high vowel with a rise of ≤ 11st; (iiia) Among Mandarin listeners, minimum duration required to identify falling tones on the high vowel [i] is longer than that on the low vowel [a] when the fall is ≤ 8st; and (iiib) a longer minimum duration required to perceive rising tones on the high vowel [i] than on the low vowel [a] for the rise of ≤ 9st.

In sum, the above results suggest that the time required to perceive pitch direction is likely to be shorter than the time required to produce them in most cases. However, times required to perceive both pitch directions mimic those needed for their production. That is, in most cases, a rising pitch requires more time to both produce and perceive. Finally, linguistic background and vowel quality play a role in the amount of time required for perception of pitch direction.

### Same-different discrimination task

For the same-different discrimination task, the collected raw data were transformed into a response of either 0 or 1 to fit generalized linear mixed models to test the significance of main effects and interaction terms. The main effects of vowel quality (χ^2^(1) = 22.59, p < 0.001), pitch direction (χ^2^(1) = 134.97, p < 0.001), and duration (χ^2^(1) = 2510.4, p < 0.001) significantly contributed to the responses of “same” or “different”. Although the main effect of language group was not significant, the two-way interaction between language group and duration showed significance (χ^2^(1) = 11.38, p < 0.001). The two-way interaction of vowel quality and duration reached marginal significance (χ^2^(1) = 3.83, p = 0.05).

#### Between-category discrimination

As mentioned in the data analysis section, we calculated d-prime scores of the comparison unit corresponding to the category boundary for each subject. Then we fit linear mixed models with d-prime scores as the response variable, and language group, pitch direction, vowel quality, and duration as factors. The subjects were included as a random effect. By likelihood ratio tests, the main effects of language group (χ^2^(1) = 12.78, p < 0.001) and duration (χ^2^(1) = 117.92, p < 0.001) reached significance. The two-way interaction between language group and duration (χ^2^(1) = 4.86, p = 0.03) and between pitch direction and duration (χ^2^(1) = 4.62, p = 0.03) were also significant.

A pairwise comparison of duration values revealed significant differences in 21% of the pairs with a duration difference of 20ms, 57% with a difference of 40ms, 62% of the pairs with a difference of 60ms, and 75% with a difference of 80ms. The results indicated that most pairs with a duration difference greater than 40ms were significantly different.

Due to significance in the language group effect, we fit two regression models for each language group separately to capture the relationship between d-prime scores and duration. For native Chinese listeners, a regression model with a quadratic term did not differ from a model without it, indicated by a likelihood ratio test (χ^2^(1) = 1.60, p = 0.21). Specifically, the formula for the relationship between the between-category d-prime scores (*bcd*) and duration (*d*) is listed as Eq 27, and the regression model was statistically significant (F(1, 26) = 47.73, p < 0.001; adjusted R^2^ = 0.63).

bcd=15.01*d−0.23 (27)

For native English listeners, the regression model suggested that duration did not significantly contribute to the change of the d-prime scores (t(6) = 1.8, p = 0.12).

Post-hoc analyses showed that the language group effects were significant for all duration values, except for the FCI vs. FEI pair with a duration of 200ms. Moreover, native Chinese listeners showed more differences (i.e., at more stimulus duration values) in perceiving rising vs. falling pitch direction than native English listeners.

#### Within-category discrimination

The d-prime scores of within-category discrimination for each subject were also calculated. Results of a linear mixed effects model suggested that the main effects of pitch direction (χ^2^(1) = 83.07, p < 0.001), duration (χ^2^(1) = 76.96, p < 0.001), and vowel quality (χ^2^(1) = 5.96, p = 0.01) significantly contributed to the d-prime scores. No interaction terms were significant.

A pairwise comparison of duration values reached significance for 7% of the pairs with a difference of 20ms, 14% of the pairs with a difference of 40ms, 8% of the pairs with a difference of 60ms, 58% of the pairs with a difference of 80ms, and 100% of the pairs with a difference of 100ms. These results indicated that many pairs did show significant difference when there was a difference greater than 80ms.

We fit two regression models for both language groups. For native Chinese listeners, a regression model with a quadratic term did not improve much, indicated by a likelihood ratio test (χ^2^(1) = 0, p = 0.99). Specifically, the formula for the relationship between the within-category d-prime scores (*wcd*) and duration (*d*) is in Eq (28), and the regression model was significant (F(1, 26) = 13.93, p = 0.009; adjusted R^2^ = 0.32).

wcd=6.20*d−0.32 (28)

For native English listeners, the regression model suggested that duration did not significantly contribute to the change of the d-prime scores (t(6) = 1.53, p = 0.18).

Compared to between-category discrimination, within-category discrimination requires larger duration differences in the stimuli for a significant difference in discrimination. For both between-category and within-category discrimination, native Chinese listeners show a significant contribution of duration, but native English listeners do not. When the duration of stimuli increases, the discrimination d-prime score increases more rapidly for between-category discrimination than within-category discrimination.

#### Peakedness

The peakedness of the discrimination function was estimated by the difference between P_bc_ (between-category discrimination) and P_wc_ (within-category discrimination). Accordingly, the d-prime scores of peakedness were calculated for each subgroup as mentioned in the data analysis section. The main effects of pitch direction (χ^2^(1) = 21.68, p < 0.001), language group (χ^2^(1) = 31.27, p < 0.001) and duration (χ^2^(1) = 12.58, p < 0.001) were significant. The interaction term between language group and duration (χ^2^(1) = 7.81, p = 0.005) was also significant.

A pairwise comparison of duration indicated significant differences for 7% of the pairs with a difference of 20ms, 33% of the pairs with a difference of 40ms, 56% of the pairs with a difference of 60ms, 58% of the pairs with a difference of 80ms, and 37% of the pairs with a difference of 100ms, which indicated that many pairs did show significant difference when there was a difference greater than 60ms. The post-hoc analyses on the interaction of group effects and duration indicated significant differences between FCI and FEI in the duration 200ms (χ^2^(1) = 4.51, p = 0.03)

Due to significant language group effects, we also fit two regression models for both language groups. For native Chinese listeners, a regression model with a quadratic term did not improve significantly by a likelihood ratio test (χ^2^(1) = 1.37, p = 0.24). Specifically, the formula for the relationship between the peakedness (*pk*) and duration (*d*) is in Eq (29), and the regression model is significant (F(1, 26) = 14.02, p = 0.009; adjusted R^2^ = 0.33).

pk=8.82*d−0.55 (29)

For native English listeners, the regression model suggested that duration did not significantly contribute to the change of peakedness (t(6) = -0.82, p = 0.44).

#### Predicted and obtained discrimination

Correlations and distances between obtained discrimination and predicted discrimination were calculated. For correlation, a linear model indicated that the main effect of pitch direction (t(45) = 2.67, p = 0.01) and the interaction between language group and pitch direction (t(45) = 2.66, p = 0.01) as well as between pitch direction and duration (t(45) = -1.791, p = 0.08) reached significance or marginal significance. Post-hoc analysis showed that the pitch direction (F vs. R) reached significance or marginal significance for all duration values except for duration values of 200ms. Rising pitch directions have a higher mean correlation than falling pitch directions. In addition, three pairs of FC vs. RC (RC > FC, χ^2^(1) = 6.95, p = 0.008), FE vs. RE (RE > FE, χ^2^(1) = 22.4, p < 0.001) and FE vs. RC (RC > FE, χ^2^(1) = 36.20, p < 0.001) reached significance.

For the distance P-P*, a linear model suggested that the main effect of pitch direction (t(275) = -2.29, p = 0.02) and the interaction between group and pitch direction (t(275) = -2.54, p = 0.01) reached significance. Rising directions have a smaller mean distance than falling pitch directions. In addition, the main effect of language group (t(275) = 1.73, p = 0.08) and duration (t(275) = 1.76, p = 0.08) reached marginal significance. Post-hoc analyses indicated that two pairs FE vs. RE (FE > RE, χ^2^(1) = 41.38, p <0.001) and FC vs. RE (FC > RE, χ^2^(1) = 13.47, p < 0.001) reached significance.

**Fig 8:**
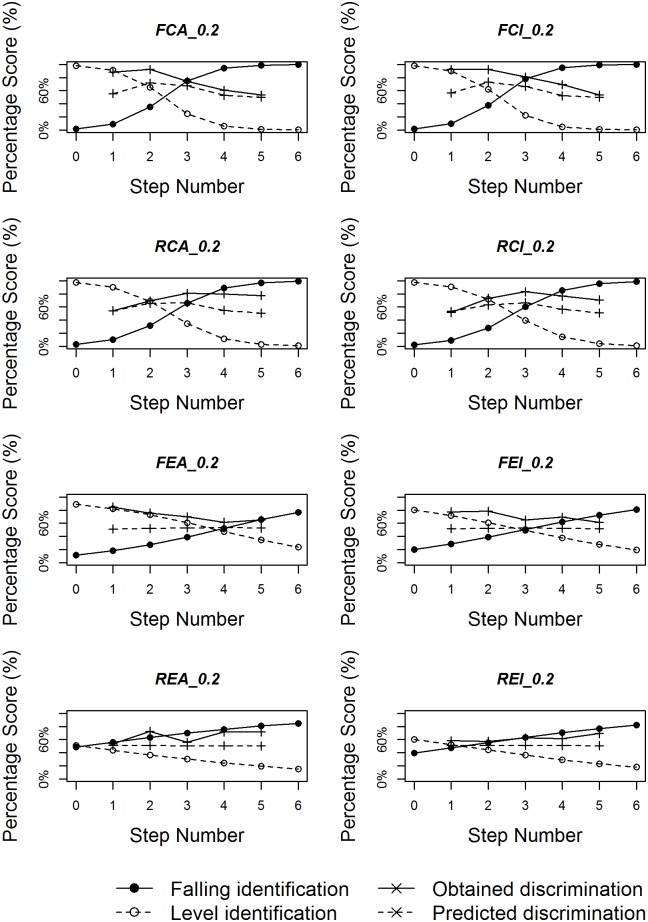
Logistic identification functions and discrimination curves for eight subgroups with duration 200ms.

**Fig 9:**
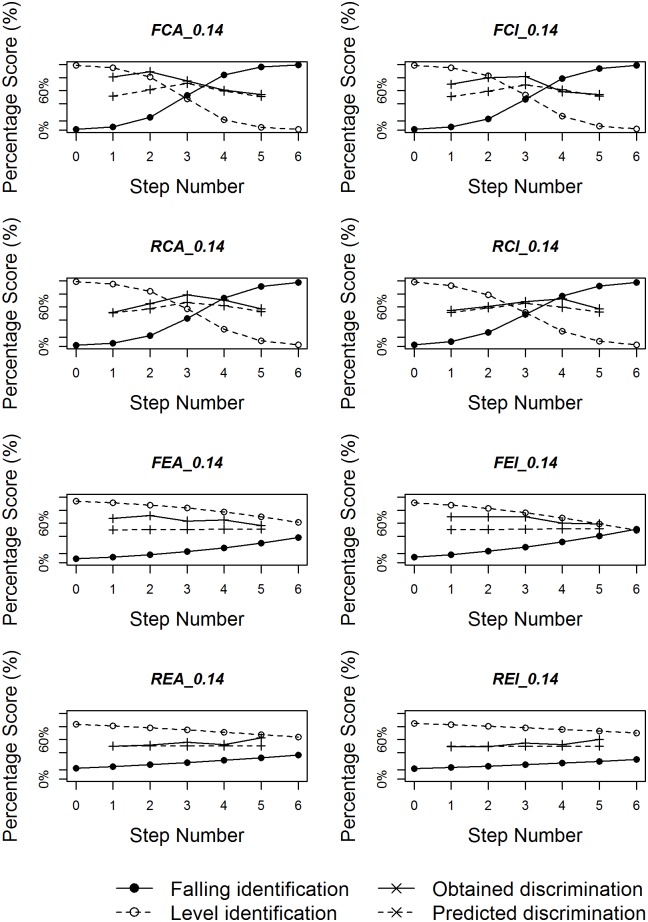
Logistic identification functions and discrimination curves for eight subgroups with duration 140ms.

**Fig 10:**
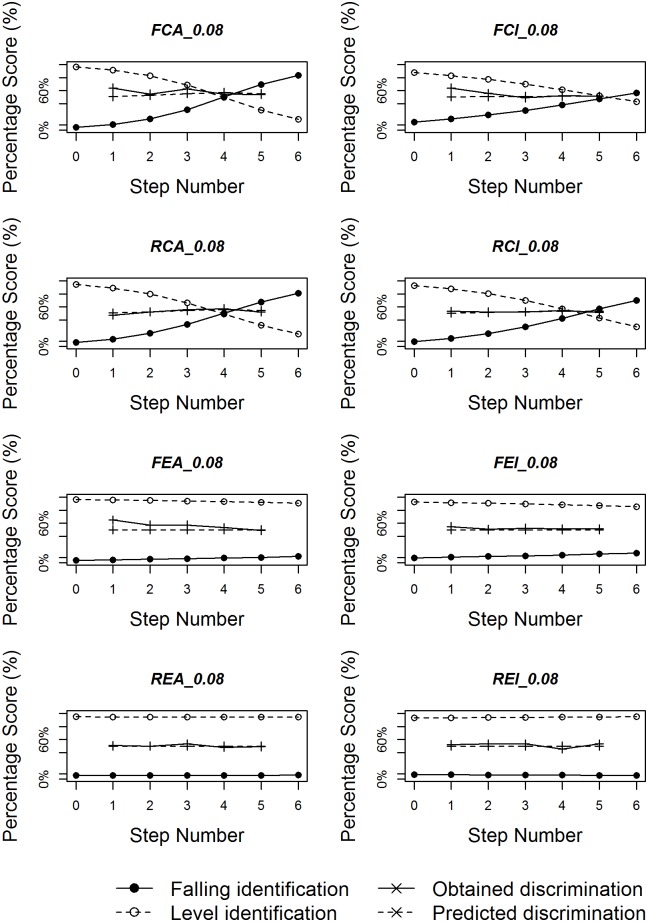
Logistic identification functions and discrimination curves for eight subgroups with duration 80ms.

Figs 8–10 plot identification functions as well as obtained and predicted discrimination of the eight subgroups with three duration values as an example, based on all the statistical analyses. For all three duration values, the figures confirm that native Chinese listeners showed stronger categorical perception than English listeners. As the duration of stimuli decreases, the categorical perception becomes weaker, with less sharp category boundary for both language groups. The correlation between obtained and predicted discrimination is also higher for native Chinese listeners than for native English listeners for most duration values.

## Summary and discussion

In this study, we aim to determine minimum duration needed by tonal and non-tonal language listeners to perceive pitch falling and rising directions, and to compare it to production results obtained by a previous study [5], to examine the effects of stimulus duration on the categorical perception of pitch directions among the two groups of listeners, and to investigate the relationship between intrinsic F0 (henceforth IF0) effect and stimulus duration on pitch perception.

### The minimum duration required for production and perception of pitch direction

We compared the formulas proposed in [5] for production of pitch directions with our formulas for pitch perception based on the data from native Chinese and English speakers and found that a minimum duration required to effectively perceive pitch directions is shorter than to produce them in many cases. According to Janse, speakers can hardly double their normal articulation rate, but have no problem in perceiving twice accelerated speech, and that a greater amount of time required for articulatory movement, motor command or speech planning [77]. Physical constraints appear to affect speech production than perceptual constraints do on speech perception. However, this is true only when the rise is greater than 1st, and the fall is greater than 6 st, suggesting the presence of different thresholds for the processing of the two pitch contours in our auditory system. A smaller threshold for a pitch rise (1st), which requires a longer duration to produce than a pitch fall, suggests that that the more difficult it is to produce, the earlier the perceptual advantage begins.

In addition, we found that relative minimum durations required to perceive the two pitch contours mimic those found for their production: longer duration is required to perceive a rising pitch than a falling pitch, thus suggesting a lack of perceptual compensation for the production of the two pitch contours.

When the two language groups were compared, contrary to what’s found in [5] that native Chinese speakers were surprisingly slower in produce a pitch direction change than native English speakers, we found that the minimum duration needed to perceive a change of 1st is longer for English listeners than for Mandarin listeners. Further calculations for the durations needed to perceive a change of 1 to 20st by the two language groups indicated that English listeners required longer duration to perceive a rising pitch direction (< 11st) and a falling pitch direction (< 12st) than tonal Chinese listeners. Therefore, there was a discrepancy between results from the speech production study and our perception results, where tonal speakers required lesser perception time, but longer production time for a given range of st. Native experience with lexical tones may heighten their perceptual sensitivity to a pitch direction change within this range. Nonetheless, the asymmetry between production and perception results deserves further attention.

### Categorical perception of tones

Regarding categorical perception, several findings clearly suggest the effects of native experience with a tonal language on pitch direction perception. First, native Mandarin listeners are more sensitive to pitch movement than American English listeners. They are more likely to identify a stimulus as having a rising or a falling pitch (as opposed to level) than American English listeners. Our result is consistent with the recent finding that the Just-Noticeable-Difference (JND) for pitch direction is smaller for Mandarin than for English listeners [78]. Second, consistent with previous studies [23, 35, 36], Mandarin listeners exhibited stronger characteristics of categorical perception of pitch contours [36]. Specifically, they showed a sharper category boundary, a higher discrimination peak, and a stronger predictability between identification and discrimination than the native English listeners. Third, Mandarin listeners’ category boundary is relatively less affected by stimulus duration. For both groups of listeners, category boundaries start earlier as the duration of the stimulus is lengthened. However, the shifting rate is slower among Mandarin listeners, thus a relatively more stable category boundary location with increased stimulus duration. These results suggest that Mandarin listeners’ identification and discrimination of pitch contours are largely mediated by their long-term phonemic memory of lexical tone categories, and to a lesser extent by auditory memory of stimulus characteristics. On the other hand, a lesser degree of categoricalness exhibited by the English listeners appears to reflect auditory rather than phonetic identification and discrimination.

In general, duration plays an important role in pitch contour perception, though the degree of the effect differs for listeners with different linguistic backgrounds. For both groups, sharpness of category boundary increased as stimulus duration increased, suggesting that pitch contour perception becomes easier with longer stimulus duration regardless of language background, suggesting a language-general effect of duration in auditory processing. In addition, a duration difference of greater than 20ms led to significant differences in the sharpness of the category boundary. Moreover, between-category discrimination would significantly differ when stimulus duration difference was greater than 40ms. Within-category discrimination was also largely affected when a duration difference reached more than 80ms. Additionally, peakedness was most strongly affected when there was a duration difference of more than 60ms. As proposed by cue-duration hypothesis [50], stimulus duration may strengthen or weaken categorical perception. Our results also clearly suggest the influence of stimulus duration on the strength of categorical perception. The fact that native Chinese listeners showed a faster rate of increase in category boundary sharpness with longer duration as well as a significantly stronger effect due to duration lengthening on between- and within- category discrimination and peakedness than native English listeners suggests that long-term experience with a tone language facilitated pitch contour perception. Long-term categorical representations could also enhance encoding of short-term categorical memory as well as being activated for “bottom-up matching of similar features” [36] (p. 1071).

More interestingly, though the dual-process model can explain the effects of linguistic background on categorical perception of pitch directions, namely the sharper category boundary and higher discrimination peak in Mandarin listeners than American listeners, suggesting phonetic memory may play a role in categorical perception, the fact that categorical perception is stronger with longer duration among Mandarin listeners is inconsistent with the cue-duration hypothesis [50]. According to the hypothesis, weak auditory memory representations of short acoustic properties such as formant transitions for stop consonants lead to or strengthen categorical perception. Although the hypothesis was supported by some studies [50, 79], where longer vowels were perceived more continuously than shorter vowels, our results on pitch directions yield the opposite relationship, with longer stimulus duration eliciting sharper category boundary and higher between- and within- category discrimination, particularly among native Mandarin listeners. As suggested by an anonymous reviewer, the acoustic information in vowel such as formants is relatively steady whereas acoustic information in tones such as F0 contour is more dynamic. This steady vs. dynamic acoustic may be responsible for the contrasting effects of stimulus duration on vowel and pitch direction perception. It is interesting to note that vowels [50] and level tones [80] seem to be perceived more continuously in a similar way. This is not surprising since F0 pattern is steadier in a level tone than in a contour tone. Our results suggest that dynamic tones are perceived more categorically by tonal listeners, and the effects of stimulus duration on the strength of categorical perception of pitch direction is the opposite of vowel perception. In future research, it is worth exploring whether stimulus duration would affect categorical perception of level tones and vowels in a similar manner.

The alternative psychoacoustic view that category boundaries reflect innate thresholds seems to be limited in explaining our results, since we found that the category boundary was also influenced by linguistic background and stimulus duration. Our results may be accounted for by multistore model of categorical perception [36]. As the stimuli duration increases, native Mandarin listeners have more time for context-coding, including matching features to their long-term representations, and encoding short-term categorical memory from long-term memory. Native English listeners cannot effectively take advantage of an increased processing time afforded by longer stimulus duration due to their lack of long-term representations of tonal categories. Alternatively, it is possible that due to Mandarin listeners’ long-term experience with pitch processing, the auditory trace of pitch information is relatively more salient and decays more slowly, and thus remains available for a longer duration to mediate phonetic labeling and discrimination among Mandarin listeners in comparison to native English listeners with relatively poorer and faster-decaying auditory memory of pitch. Additional research involving listeners from different tonal language backgrounds is needed to further explore these hypotheses.

Finally, the category boundary was found to be sharper for a falling than a rising pitch contour for both groups of listeners. This is consistent with a recent report [1] that native Chinese speakers processed English words produced with a falling F0 more quickly than those produced with a rising F0. English speakers also show the same pattern, which suggests that a falling contour may be more psychoacoustically salient than a rising contour. This explanation is consistent with the data obtained in [81], showing perceptual advantages for falling over rising contours when discriminated against a preceding level contour (LF versus LR). However, both Mandarin and English listeners found a rising contour to be more discriminable than a falling contour from a following level contour (FL versus FR) in that study. Support for greater perceptual saliency of rising contour over falling contour is also suggested by the finding that brain stem’s frequency-following-response (FFR) was more faithful and robust for rising pitch contour (Mandarin Tones 2 and 3) than for level and falling pitch contours (Mandarin Tone 1 and 4) [82]. It is possible that categorizability of pitch contour depends, not on its perceptual saliency, but on the discontinuity of the auditory system (where discrimination is enhanced in those natural boundaries of perceptual space [83]. Further research is needed to explore this relationship.

### The interaction between vowel quality and duration

Previous studies mainly focus on whether the perceived duration of vowels is related to pitch contours. For example, Yu examined how the spectral and F0 effects interact in the perception of vowel duration [62]. Although in speech production, high vowels correlate with higher F0 values, whereas low vowels with lower F0 values in general, perceptual compensation can occur where F0 values are judged to be lower for higher vowels than for low vowels even if they are acoustically the same. Yu hypothesized that if perceptual compensation occurs for high vowels, then high vowels may be perceived as longer [62]. Instead, our study varied the stimulus duration to examine how tone perception is affected. Based on our results, vowel quality plays a limited role in categorical perception since it only affects within-category discrimination, but not the sharpness of category boundary, the location of category boundary, between-category discrimination, or peakedness.

Our results rejected the null hypothesis that the same duration is needed for effective perception of high and low vowels. Instead, we found that in general, for both Mandarin and English listeners, both pitch directions required a longer duration to perceive in the low vowel context than in the high vowel context. The only exception was the Mandarin listeners’ perception of the falling pitch contour: a longer time was needed to perceive a pitch fall of less than 9st produced on a high vowel than on a low vowel. Therefore, perception of a falling pitch contour can be best described by Fig 1(b), where its offset value on a low vowel are perceived to be higher than high vowels, thus a shallower slope and a longer duration required to differentiate it from a level contour. However, for a rising pitch direction, the perception was similar to the situation described in Fig 2(c) with no significant difference between low and high vowels. Yu also discovered that vocalic compensation is attenuated when the syllable carries a rising tone, which suggests that vowel quality may not play a critical role in the perceived duration of rising tones [62]. The attenuation is also reflected in our results, where the low and high vowels showed similar perception time on a rising, but not on a falling pitch direction.

## Conclusions

Perception of pitch contour is affected by language experience, stimulus duration, and, to a lesser extent, vowel quality. First, tone-language listeners are more sensitive to pitch movement and exhibit stronger categorical perception of pitch direction than non-tone language listeners. Second, stimulus duration affects tone- and non-tone language listeners’ perception of pitch contour to different degrees. Category boundary sharpness increases with longer stimulus duration, but at a faster rate among tone- compared to non-tone language listeners. Additionally, a stronger duration effect was found for tone-language listeners’ between- and within- category discrimination and categorical peakedness, suggesting facilitative effects of longer stimulus duration on context-coding, encoding short-term categorical memory from their long-term memory. Third, for both groups of listeners, a sharper category boundary was found for falling than for rising pitch directions. Fourth, a relatively shorter duration is required to perceive than to produce pitch contours, and non-tonal listeners need a longer duration to detect a change in pitch movement within a certain range of st than tonal listeners. Finally, vowel quality only affects perception of a falling pitch contour.

## Supporting information

S1 Table Pairwise comparisons between different duration values within eight subgroups. (ZIP)
