# Supplementary material for: Effects of stimulus duration and vowel quality in cross-linguistic categorical perception of pitch directions
Source: PLoS One. 2017 Jul 3;12(7):e0180656. doi: 10.1371/journal.pone.0180656 (PMC5495489; doi:10.1371/journal.pone.0180656)
Supplement: S1 Table — (ZIP) [file pone.0180656.s001.zip › supporting information/Supporting information.docx]

**Supporting Information**

**S1 Table. Pairwise comparisons between different duration values within eight subgroups**

| **Subgroup** | **Pairs of duration** | **Likelihood ratio tests** |
| --- | --- | --- |
| **FCA** | 0.18 vs. 0.2 | χ^2^(1) = 0.93, p = 0.33 |
|  | 0.14 vs. 0.16 | χ^2^(1) = 3.62, p = 0.06 |
|  | 0.12 vs. 0.14 | χ^2^(1) = 1.37, p = 0.24 |
|  | 0.04 vs. 0.06 | χ^2^(1) = 0.50, p = 0.48 |
| **FCI** | 0.16 vs. 0.18 | χ^2^(1) = 0.90, p = 0.34 |
|  | 0.1 vs. 0.12 | χ^2^(1) = 0.003, p = 0.96 |
|  | 0.06 vs. 0.08 | χ^2^(1) = 2.92, p = 0.087 |
|  | 0.04 vs. 0.08 | χ^2^(1) = 0.39, p = 0.53 |
| **RCA** | 0.16 vs. 0.18 | χ^2^(1) = 0.2856, p = 0.5931 |
|  | 0.14 vs. 0.18 | χ^2^(1) = 1.4197, p = 0.2335 |
|  | 0.1 vs. 0.18 | χ^2^(1) = 0.2353, p =0.6276 |
|  | 0.14 vs. 0.16 | χ^2^(1) = 0.3858, p = 0.5345 |
|  | 0.1 vs. 0.16 | χ^2^(1) = 0.9261, p = 0.3359 |
|  | 0.12 vs. 0.14 | χ^2^(1) = 2.5786, p = 0.1083 |
|  | 0.1 vs. 0.14 | χ^2^(1) =2.54 , p = 0.111 |
| **RCI** | 0.16 vs. 0.2 | χ^2^(1) =2.4288 , p =0.1191 |
|  | 0.16 vs. 0.18 | χ^2^(1) = 0.7941, p = 0.3729 |
|  | 0.14 vs. 0.18 | χ^2^(1) = 0.0291, p =0.8647 |
|  | 0.14 vs. 0.16 | χ^2^(1) = 0.4944, p = 0.482 |
| **FEA** | 0.1 vs. 0.12 | χ^2^(1) = 2.7312, p =0.0984 |
|  | 0.08 vs. 0.1 | χ^2^(1) = 0.2991, p =0.5844 |
|  | 0.06 vs. 0.1 | χ^2^(1) = 2.3431, p = 0.1258 |
|  | 0.06 vs. 0.08 | χ^2^(1) = 1.0278, p = 0.31 |
|  | 0.04 vs. 0.08 | χ^2^(1) = 2.6088, p = 0.11 |
|  | 0.04 vs. 0.06 | χ^2^(1) = 0.3951, p = 0.5296 |
| **FEI** | 0.16 vs. 0.18 | χ^2^(1) = 0.1147, p = 0.7349 |
|  | 0.12 vs. 0.14 | χ^2^(1) = 2.7654, p = 0.09632 |
|  | 0.1 vs. 0.12 | χ^2^(1) = 0.1219, p = 0.7269 |
|  | 0.06 vs. 0.08 | χ^2^(1) = 0.5088, p = 0.4757 |
|  | 0.04 vs. 0.06 | χ^2^(1) = 2.3085 , p = 0.1287 |
| **REA** | 0.1 vs. 0.12 | χ^2^(1) = 2.3192, p = 0.1278 |
|  | 0.06 vs. 0.08 | χ^2^(1) = 3.563, p = 0.05908 |
|  | 0.04 vs. 0.08 | χ^2^(1) = 0.1945, p = 0.6592 |
|  | 0.04 vs. 0.06 | χ^2^(1) = 2.1538, p =0.1422 |
| **REI** | 0.16 vs. 0.18 | χ^2^(1) = 3.5677, p = 0.05891 |
|  | 0.06 vs. 0.08 | χ^2^(1) = 1.7277, p = 0.1887 |
|  | 0.04 vs. 0.08 | χ^2^(1) = 0.283, p = 0.5947 |
|  | 0.04 vs. 0.06 | χ^2^(1) = 0.5962, p = 0.44 |
| F: Falling; R: Rising; C: Chinese; E: English; A: Vowel [a]; I: Vowel [i] | | |
